# Secondary necrotic neutrophils release interleukin-16C and macrophage migration inhibitory factor from stores in the cytosol

**DOI:** 10.1038/cddiscovery.2015.56

**Published:** 2015-11-30

**Authors:** S Roth, M Agthe, S Eickhoff, S Möller, C M Karsten, N Borregaard, W Solbach, T Laskay

**Affiliations:** 1 Institute for Medical Microbiology and Hygiene, University of Lübeck, Ratzeburger Allee 160, Lübeck 23562, Germany; 2 Institute for Systemic Inflammation Research, University of Lübeck, Ratzeburger Allee 160, Lübeck 23562, Germany; 3 Department of Hematology, Granulocyte Research Laboratory, L-4041, Finsen Center, The National University Hospital, 9 Blegdamsvej, Copenhagen DK-2100, Denmark

## Abstract

Neutrophils harbor a number of preformed effector proteins that allow for immediate antimicrobial functions without the need for time-consuming *de novo* synthesis. Evidence indicates that neutrophils also contain preformed cytokines, including interleukin (IL)-1ra, CXCL8 and CXCL2. In the search for additional preformed cytokines, a cytokine array analysis identified IL-16 and macrophage migration inhibitory factor (MIF) as preformed cytokines in lysates from human primary neutrophils. Both IL-16 and MIF are unconventional cytokines because they lack a signal sequence. Using confocal immunofluorescence microscopy as well as western blot analysis of subcellular fractions, IL-16 and MIF were found to be stored in the cytosol rather than in the granules of human neutrophils, which implies an unconventional secretion mechanism for both cytokines. IL-16 is synthesized and stored as a precursor (pre-IL-16). We present evidence that the processing of pre-IL-16 to the biologically active IL-16C is mediated by caspase-3 and occurs during both spontaneous and UV-induced apoptosis of human neutrophils. Although IL-16 processing occurs during apoptosis, IL-16C and MIF release was observed only during secondary necrosis of neutrophils. Screening a panel of microbial substances and proinflammatory cytokines did not identify a stimulus that induced the release of IL-16C and MIF independent of secondary necrosis. The data presented here suggest that IL-16 and MIF are neutrophil-derived inflammatory mediators released under conditions of insufficient clearance of apoptotic neutrophils, as typically occurs at sites of infection and autoimmunity.

## Introduction

Neutrophil granulocytes have a short lifespan. They undergo apoptosis within a few hours and are cleared from the circulation in the liver, spleen and bone marrow.^1^ At sites of infection and inflammation, their lifespan is prolonged.^2^ However, after having fulfilled their functions, large numbers of neutrophils undergo apoptosis at the site of infection/inflammation.

Apoptotic cell death is generally characterized by chromatin condensation and fragmentation, cell shrinkage, blebbing of the plasma membrane, formation of apoptotic bodies, activation of caspase-3 and presentation of ‘find-me’ and ‘eat-me’ signals. A fundamental feature of apoptotic cell death is the maintenance of membrane integrity in order to prevent leaking of toxic cellular contents.^3^ However, the integrity of the cell membrane of apoptotic neutrophils cannot be maintained for an extended period of time. Consequently, in the case of insufficient clearance, apoptotic neutrophils undergo secondary necrosis. Necrosis is defined by cell lysis, followed by the release of DAMPs (danger-associated molecular pattern molecules), which results in the activation of inflammatory and immune processes. Whereas primary necrosis is induced by highly toxic substances, leading to the swelling and consequent lysis of cells, secondary necrosis is the consequence of apoptotic cells losing their membrane integrity. Therefore, major differences exist between the release of DAMPs from cells undergoing primary necrosis and that from cells undergoing secondary necrosis. In particular, secondary necrotic cells release considerably less ATP, although they release activated caspase-3 and proteolytically processed autoantigens.^4–6^

As fast-acting effector cells of the innate immune system, neutrophils are rapidly recruited to sites of infection, where they exert their antimicrobial function.^7,8^ To enable this rapid action, neutrophils harbor preformed antimicrobial effector molecules, such as defensins, lysozyme and cathelicidins, which can act immediately after cell activation without a need for time-consuming *de novo* synthesis.^9,10^ Therefore, many of the preformed substances are antimicrobial effector molecules. In addition, neutrophils also contain preformed cytokines, including CXCL8^11^ and CXCL2,^12^ which have important roles in the rapid recruitment of inflammatory cells to sites of injury or infection.

In the present study, we searched for additional preformed mediators of inflammation and identified interleukin (IL)-16 and macrophage migration inhibitory factor (MIF) as preformed cytokines in primary human neutrophils. Western blot analysis and confocal microscopy revealed that both IL-16 and MIF are stored in the cytosol rather than in neutrophil granules. We showed that IL-16 is processed in a caspase-3-dependent manner in apoptotic neutrophils, giving rise to the biologically active C-terminal fragment, IL-16C. Importantly, the release of both IL-16 and MIF correlates strongly with the secondary necrosis of neutrophils. We were not able to identify any stimuli that induced the release of IL-16 and MIF independent of neutrophil secondary necrosis. Therefore, IL-16 and MIF represent potential mediators and modulators of inflammatory and immune responses at sites of insufficient clearance of apoptotic neutrophils.

## Results

### IL-16 and MIF are preformed cytokines in primary human neutrophils

**Figure 1:**
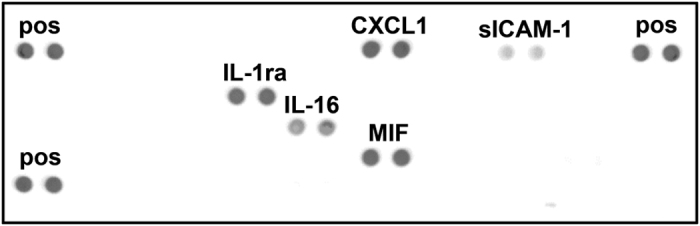
Preformed cytokines in human neutrophils. Lysate of freshly isolated neutrophils was analyzed for preformed cytokines using Human Cytokine Array Panel A, screening 36 cytokines, chemokines and acute-phase proteins. pos, positive control.

Mature neutrophils contain several preformed antimicrobial proteins.^13^ In addition, some cytokines have been shown to be stored in mature neutrophils.^11,12,14–20^ To obtain broader insight into the preformed cytokines of human neutrophils, a lysate from freshly isolated primary human neutrophils was analyzed using the Proteome Profiler Human Cytokine Array Kit (R&D Systems, Minneapolis, MN, USA). Out of the 36 cytokines, chemokines and acute-phase proteins screened, positive signals were obtained for the cytokines IL-1ra, IL-16, CXCL1 and MIF, indicating the intracellular presence of these cytokines in resting human neutrophils (Figure 1). In addition, a signal for sICAM-1 was detected (Figure 1). However, because a cell lysate rather than cell culture supernatant was analyzed with the Cytokine Array Kit in our study, the sICAM-1 signal likely indicates the presence of ICAM-1 in the neutrophil cell membrane rather than the soluble form of ICAM-1.

### Preformed IL-16 and MIF are localized in the cytosol of human neutrophils

Antimicrobial proteins represent the majority of preformed proteins in neutrophils. They are formed during granulopoiesis and stored in neutrophil granules.^21^ Neutrophils possess at least four different types of granules: (i) azurophilic/primary granules, (ii) specific/secondary granules, (iii) gelatinase/tertiary granules and (iv) secretory vesicles. The various granule populations contain distinct sets of proteins, some of which are used as identification markers for the particular granule type.

**Figure 2:**
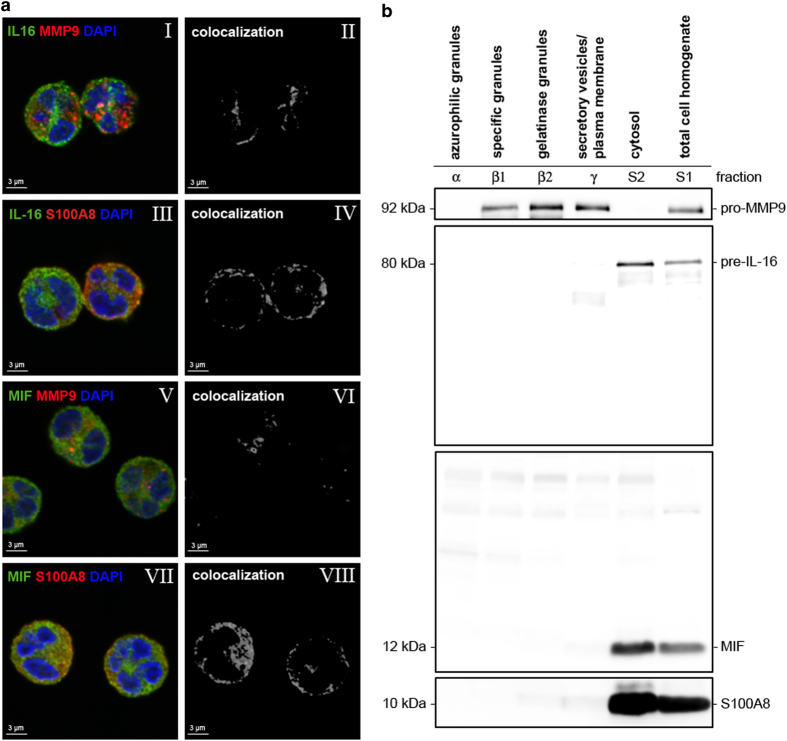
Cytosolic storage of IL-16 and MIF in neutrophils. (**a**) Neutrophils were fixed and stained for IL-16 (green, I and III), MIF (green, V and VII), MMP9 (red, I and V), S100A8 (red, III and VII) and the nuclei (DAPI, blue; I, III, V and VII). Staining was analyzed using confocal microscopy. Colocalization studies of the fluorescent signals for IL-16 and MMP9 (II), IL-16 and S100A8 (IV), MIF and MMP9 (VI), MIF and S100A8 (VIII) were performed. White signals indicate colocalization of the green and red fluorescent signals. Bars, 3 *μ*m. The presented data are representative of three independent experiments. (**b**) Freshly isolated neutrophils were fractionated in a two-step Percoll gradient and analyzed for its content of IL-16, MIF, pro-MMP9 and S100A8 using western blot analysis. The presented data are representative of two experiments.

IL-16 and MIF share a feature that is rather uncommon for cytokines: they both lack a signal sequence.^22,23^ Having observed that these two cytokines with this unusual feature are present in neutrophils, we addressed the issue of the subcellular localization and release of IL-16 and MIF by primary human neutrophils *in vitro* by applying immunofluorescence staining and confocal fluorescence microscopy. Imaging confirmed the intracellular presence of preformed IL-16 and MIF in neutrophils (Figure 2a). The white spots/areas shown represent the results of the computer-assisted analysis of colocalization of the green IL-16 signals with the red signals of S100A8 (Figure 2a, III and IV). The analysis showed a strong colocalization of IL-16 with S100A8, an abundant cytosolic protein in neutrophils (Figure 2a, IV). In contrast, colocalization analysis of IL-16 with gelatinase (MMP9; Figure 2a, I and II), a protein contained in both gelatinase and specific granules, showed a weak colocalization with IL-16. Similarly, a very strong colocalization between MIF and S100A8 signals was also observed (Figure 2a, VII and VIII), whereas only very weak colocalization was observed between the MIF and MMP9 signals (Figure 2a, V and VI).

The data thus indicate that IL-16 and MIF are stored in the cytosol rather than in the granules of neutrophils. To confirm this finding, subcellular fractions of neutrophils were analyzed using western blot analysis. The total cell homogenate as well as five subcellular fractions, that is, azurophilic granules, specific granules, gelatinase granules, secretory vesicles/plasma membrane and cytosol, were assessed for the presence of IL-16 and MIF. This analysis showed that IL-16 and MIF are present in the cytosol rather than in any of the granules or vesicles (Figure 2b). Therefore, this analysis clearly confirms the intracellular presence and cytosolic localization of both IL-16 and MIF in primary human neutrophils.

### pre-IL-16 is processed in apoptotic neutrophils in a caspase-3-dependent manner

IL-16 is synthesized as an ~80-kDa precursor molecule,^24^ recently termed pre-IL-16.^25^ Previously, it was shown in CD4^+^ and CD8^+^ T cells that pre-IL-16 is cleaved enzymatically by caspase-3,^23,26^ a cysteine–aspartic acid protease that has a major role in apoptosis. Caspase-3-mediated processing of pre-IL-16 results in the generation of the 17-kDa peptide IL-16C and the larger fragment pro-IL-16.^23^ IL-16C is the secreted fragment, exerting chemotactic activity on CD4^+^ cells.^27,28^

Neutrophils undergo spontaneous apoptosis within 40 h when cultured *in vitro*.^29^ To investigate whether pre-IL-16 is cleaved into the biologically active IL-16C during neutrophil apoptosis, neutrophils were analyzed after 5, 10, 20, 30, 40 and 60 h of incubation *in vitro*. The proportions of apoptotic cells were assessed by staining cells with Annexin-V-FLUOS and propidium iodide (PI). The intracellular levels of pre-IL-16 and IL-16C were assessed using western blot analysis.

**Figure 3:**
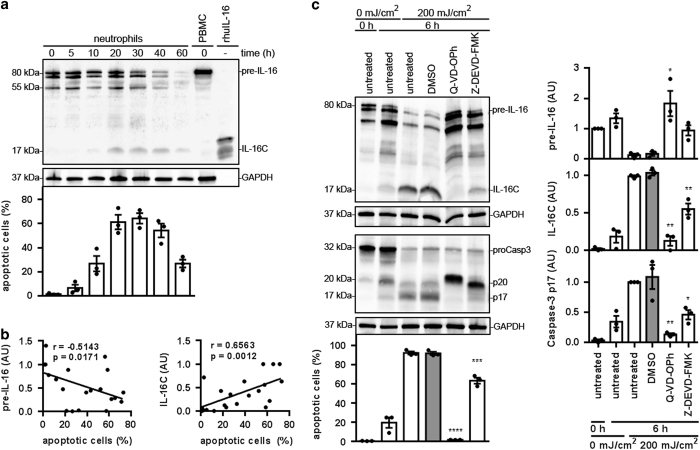
Caspase-3-mediated processing of pre-IL-16 in apoptotic neutrophils. (**a** and **b**) Neutrophils were cultured for 0, 5, 10, 20, 30, 40 and 60 h at 37 °C. (**a**) Lysates were prepared at the indicated time points and analyzed for IL-16 and GAPDH (loading control) using western blot analysis. Shown are representative blots of three independent experiments. Neutrophil viability was assessed with flow cytometry using Annexin-V-FLUOS and PI staining. Bars represent the mean of the proportion of apoptotic cells (Annexin-V-FLUOS^+^/PI^−^)±S.E.M. of three independent experiments. Individual data points are shown as black dots. (**b**) The correlation between pre-IL-16 (left)/IL-16C (right) levels (normalized to GAPDH) and the proportion of apoptotic cells. The data were analyzed by the nonparametric Spearman correlation, *n*=21, *P* (two-tailed). (**c**) Neutrophils were irradiated with 200 mJ/cm^2^ UV light (256 nm). Immediately after radiation, the neutrophils were treated with the pan-caspase inhibitor (Q-VD-OPh, 1 *μ*M), the caspase-3-specific inhibitor (Z-DEVD-FMK, 100 *μ*M) and the solvent DMSO (1 : 200). Lysates were prepared after 6 h of incubation at 37 °C and analyzed for IL-16, caspase-3 and GAPDH (loading control) using western blot analysis. Shown are representative blots of three independent experiments (left panel), which were quantified and normalized to GAPDH (right panel). Neutrophil viability was assessed by flow cytometry using Annexin V-FLUOS and PI staining. Bars represent the mean of the percentage of apoptotic cells (Annexin V-FLUOS^+^/PI^−^)±S.E.M. of three independent experiments (*P<0.05; **P<0.01; ***P<0.001; ****P<0.0001 compared with the DMSO sample by one-way ANOVA followed by Holm Sidak multiple comparison correction). Individual data points are shown as black dots. AU, arbitrary units.

Analysis of freshly isolated neutrophils revealed a double band at 75–80 kDa and a single band at 55 kDa (Figure 3a, Supplementary Information and Supplementary Figure 1). The intensities of all three bands decreased with increasing incubation times. After 10 h of incubation, the 17-kDa IL-16C band appeared (Figure 3a). The intensity of the IL-16C band became stronger with an increased duration of incubation. A strong negative correlation between the abundance of the non-processed pre-IL-16 band and apoptosis became evident (Figure 3b, left panel). At the same time, a strong correlation was observed between the level of the IL-16C band and neutrophil apoptosis (Figure 3b, right panel). These results indicate that pre-IL-16 is indeed processed during apoptotic cell death in neutrophils.

Our results suggest that pre-IL-16 is cleaved by caspase-3, as has been observed in T cells.^26^ To confirm this hypothesis, neutrophil apoptosis was induced by irradiation with 200 mJ/cm^2^ UV light (256 nm). Immediately after irradiation, neutrophils were treated with a pan-caspase inhibitor (Q-VD-OPh), a caspase-3-specific inhibitor (Z-DEVD-FMK) and the solvent DMSO as control. After 6 h of incubation at 37 °C, neutrophil viability was assessed with Annexin-V-FLUOS and PI staining. The intracellular levels of pre-IL-16 and IL-16C were assessed with western blot analysis.

UV light irradiation increased the apoptosis from ~20 to ~92%, inducing caspase-3 activation and pre-IL-16 processing into IL-16C after 6 h of incubation (Figure 3c). Treatment with Q-VD-OPh and Z-DEVD-FMK significantly inhibited both apoptosis and the processing of pre-IL-16 into IL-16C by blocking the autocatalytic cleavage of the p20 fragment of caspase-3 into the p17 fragment (Figure 3c). Processing and consequent activation of caspase-3 occur in a two-step process.^30^ First, pro-caspase-3 is cleaved by the upstream caspases-8 and -9 into a heterotetramer complex consisting of two p20 and two p12 fragments. In the second step, the p20 fragments are autocatalytically processed into p17 fragments, forming the mature and biologically active p17/p12-caspase-3 complex. Our results show that it is the p17/p12-caspase-3 complex that mediates IL-16 processing in neutrophils.

### IL-16C and MIF are released from neutrophils during secondary necrosis

**Figure 4:**
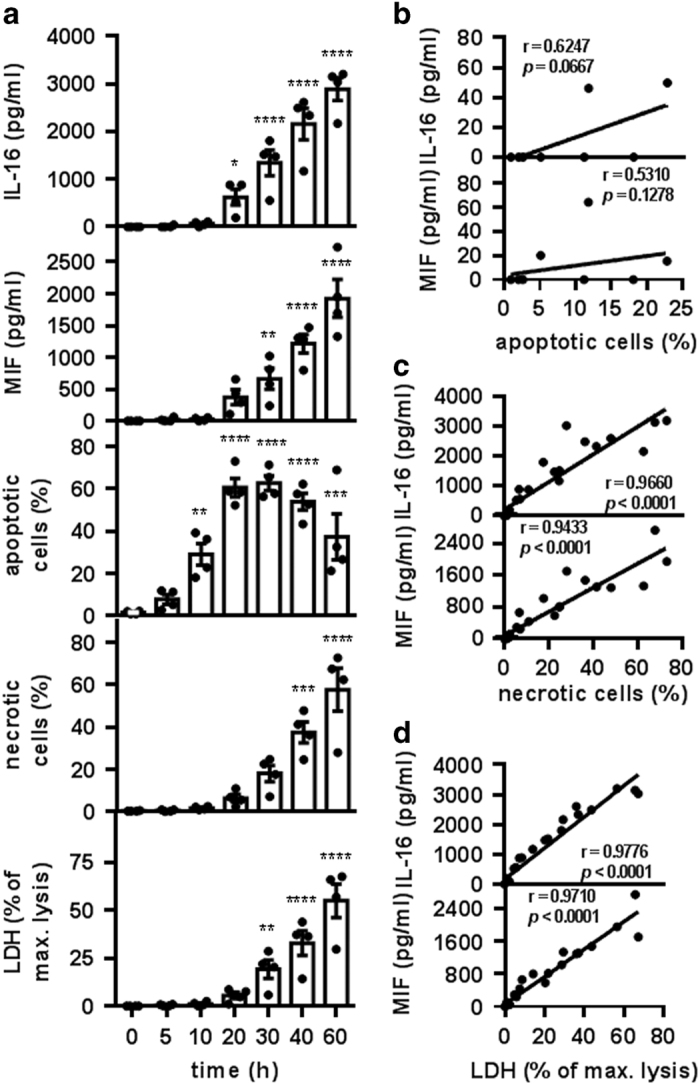
IL-16C release during secondary necrosis of neutrophils. (**a**–**d**) Neutrophils were incubated for 0, 5, 10, 20, 30, 40 and 60 h at 37 °C. (**a**) At the indicated time points, the samples were centrifuged at 400×*g* for 10 min. Supernatants were collected and centrifuged at 4 °C and 60 000×*g* for 1 h before being analyzed with ELISA for released IL-16 and MIF and with a cytotoxicity detection kit for LDH (depicted in % of max. lysis, as obtained by lysis of freshly isolated neutrophils with Triton X-100). Neutrophil viability was assessed with flow cytometry using Annexin-V-FLUOS and PI staining. Bars represent the means±S.E.M. of four independent experiments (*P<0.05; **P<0.01; ***P<0.001, ****P<0.0001 compared with the 0-h sample by one-way ANOVA followed by Holm Sidak multiple comparisons correction). Individual data points are shown as black dots. (**b**–**d**) Correlation analysis of the data obtained in **a**, analyzed by nonparametric Spearman correlation, *P* (two-tailed). (**b**) The correlation between the percentage of apoptotic neutrophils and the concentration of IL-16 (top) or MIF (bottom) in the supernatants. Samples containing more than 3% necrotic cells were excluded. *n*=10. (**c**) The correlation between the percentage of necrotic neutrophils and the concentration of IL-16 (top) or MIF (bottom) in the supernatants. *n*=28. (**d**) The correlation between the percentage of LDH release and the concentration of IL-16 (top) or MIF (bottom) in the neutrophil supernatants. *n*=28.

Because IL-16 is processed during neutrophil apoptosis (Figure 3), we addressed the question of whether the IL-16C peptide was secreted by apoptotic neutrophils. Supernatants of *in vitro* cultured neutrophils were collected at various time points during 60 h of incubation, ultracentrifuged and their content of IL-16 and MIF determined. The proportion of apoptotic and necrotic cells at the indicated time points was assessed using flow cytometry following staining with Annexin-V-FLUOS and PI. In addition, the lactate dehydrogenase (LDH) enzyme activity was detected in the cell culture supernatants as a measure of cell death-associated release of cellular content. As early as 5 h of incubation, ~10% of the cells were apoptotic; this proportion increased to ~35% after 10 h and to more than 60% after 20 h of incubation (Figure 4a). Only a small proportion of necrotic cells was detected 20 h after incubation. After that, the proportion of necrotic cells increased gradually during the 60 h of culture (Figure 4a). Similarly, LDH release was first detected after 20 h of culture. After this time point, the LDH levels of the culture supernatants increased with time, indicating enhanced cell death during prolonged *in vitro* culture (Figure 4a, top). Notably, IL-16 release did not have the same kinetics as apoptosis. Instead, IL-16 could be detected in the supernatants only after the appearance of secondary necrotic cells (Figure 4a). A very strong correlation was observed between IL-16 levels and the percentage of secondary necrotic cells as well as LDH release (Figures 4c and d, top). However, the correlation between IL-16 release and the proportion of apoptosis was notably weaker and not significant (Figure 4b, top).

Biologically active MIF does not require caspase-dependent proteolytic processing. However, in a previous study, MIF release was observed from apoptotic but not from viable neutrophils.^14^ We assessed the release of MIF from neutrophils at various time points during the 60 h of *in vitro* culture. MIF release did not follow the kinetics of apoptosis but rather the kinetics of secondary necrosis of neutrophils (Figure 4a). MIF appeared in the supernatants only when necrotic cells and LDH release were detectable. Similar to IL-16, a very strong and significant correlation was observed between the proportion of secondary necrotic cells and MIF release as well as between LDH release and MIF release (Figures 4c and d, bottom). Only a weak and nonsignificant correlation was observed between MIF release and the proportion of apoptosis (Figure 4b, bottom).

**Figure 5:**
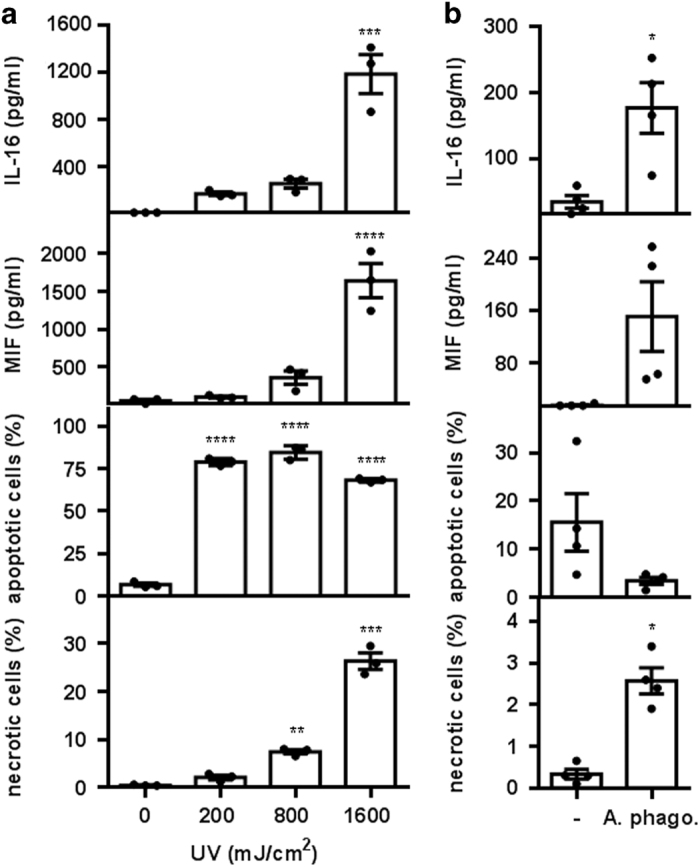
IL-16C release upon treatments inducing secondary necrosis of neutrophils. (**a**) Neutrophils were irradiated with 0, 200, 800 and 1600 mJ/cm^2^ UV light (256 nm). After 6 h of incubation at 37 °C, the supernatants were analyzed with ELISA for released IL-16 and MIF. Neutrophil viability was assessed with flow cytometry using Annexin-V-FLUOS and PI staining. Bars represent the means±S.E.M. of three independent experiments (*P<0.05; **P<0.01; ***P<0.001, ****P<0.0001 compared with the 0-h sample by one-way ANOVA followed by Holm Sidak multiple comparison correction). Individual data points are shown as black dots. (**b**) Neutrophils were infected with *A. phagocytophilum* (A. phago) for 6 at 37 °C. The supernatants were analyzed with ELISA for released IL-16 and MIF, and neutrophil viability was assessed with flow cytometry using Annexin-V-FLUOS and PI staining. Bars represent the means±S.E.M. of four independent experiments (*P<0.05; **P<0.01; ***P<0.001, ****P<0.0001 compared with the uninfected control by paired *t*-test). Individual data points are shown as black dots.

Our results revealed that IL-16C and MIF are released from secondary necrotic rather than apoptotic neutrophils. In an attempt to clarify the role of apoptosis in the release of IL-16C and MIF, we modulated neutrophil apoptosis. For this purpose, spontaneous neutrophil apoptosis was facilitated using UV-light irradiation (256 nm). UV-light irradiation enhanced neutrophil apoptosis in a dose-dependent manner (Figure 5a). Importantly, although irradiation with 200 mJ/cm^2^ UV-light-induced apoptosis in ~80% of neutrophils, no significant release of IL-16 and MIF was observed. However, irradiation with higher UV doses led to the release of high IL-16 and MIF levels (Figure 5a). Notably, increasing the UV dose enhanced the degree of apoptosis only marginally but led to the induction of secondary necrosis in ~25% of neutrophils (Figure 5a). These results demonstrate once more that IL-16C and MIF are released not from apoptotic neutrophils but from secondary necrotic neutrophils, as has been shown during spontaneous cell death.

As another approach for modulating neutrophil apoptosis, neutrophils were infected with *Anaplasma phagocytophilum*, an intracellular bacterium that preferentially infects neutrophil granulocytes. Infection with *A. phagocytophilum* has been shown to inhibit neutrophil apoptosis.^31^ As expected, after 6 h of incubation, neutrophils in the infected culture had a lower level of apoptosis (Figure 5b). Still, cells in the infected culture released significantly higher levels of IL-16 and apparently higher levels of MIF compared with uninfected neutrophils (Figure 5b). However, infected cells also contained a significantly higher proportion of necrotic neutrophils (Figure 5b), substantiating the view that the release of IL-16 and MIF is dependent on secondary necrosis rather than apoptosis.

## Discussion

Neutrophils are capable of expressing and secreting more than 50 different cytokines.^32^ A few of these, such as IL-1ra,^20^ IL-4,^17^ IL-6,^15^ CXCL8,^11^ IL-12,^16^ MIF,^14^ CXCL2,^12^ TGF-*α* ^19^ and VEGF,^33^ are already produced during granulopoiesis and form pools of preformed cytokines in mature neutrophils. As the secretion of preformed cytokines is not delayed by prior *de novo* synthesis, these cytokines are believed to have a specific role during the early phase of inflammation.

Using two gradient centrifugations and endotoxin-free reagents, we isolated neutrophils with minimal or no activation and contamination with other immune cells. In a lysate from these neutrophils, we identified the presence of CXCL1, IL-1ra, IL-16, MIF and sICAM-1 among the 36 cytokines and acute-phase proteins screened. This is in accordance with previous studies that also found IL-1ra^20^ and MIF^14^ to be preformed cytokines in human neutrophils. Moreover, CXCL2 (MIP-2), the murine homolog of CXCL1 and CXCL8, has been described to be a preformed cytokine in murine neutrophils.^12^ The results of our array now confirm that CXCL1 is also preformed in human neutrophils. In contrast to this finding, our screening did not detect IL-6 and IL-12 among the preformed cytokines in human neutrophils, although the preformed presence of these cytokines has been described in murine neutrophils.^15,16^ Furthermore, we could not confirm the intracellular storage of IL-4^17^ and CXCL8,^11^ as has been shown previously in human neutrophils. However, as these stores are supposed to contain only small amounts of IL-4 and CXCL8, the sensitivity of our array may not have been sufficient to detect these cytokines.

Although the presence of preformed IL-1ra^20^ and MIF^14^ has been described before in human neutrophils, IL-16 has not. There has even been doubt raised as to whether neutrophils express IL-16 at all.^32^ In other cells, IL-16 is synthesized as a precursor molecule (pre-IL-16), which is cleaved by caspase-3 into the secreted and biologically active IL-16C.^23,24,26^ If pre-IL-16 is a preformed cytokine in human neutrophils, it should also be processed during apoptosis. Indeed, our western blot analysis showed that pre-IL-16 is cleaved into IL-16C during apoptosis of human neutrophils. Because caspase-3 inhibitors block pre-IL-16 cleavage in human neutrophils, the processing of pre-IL-16 is dependent on caspase-3 activation and presumably does not involve other proteases. Nonetheless, it has been shown in T cells that caspase-3 activation and pre-IL-16 processing are not automatically accompanied by apoptosis. We are not aware of apoptosis-independent activation of caspase-3 in neutrophils, nor were we able to detect a constant background caspase-3 activation, as postulated for CD8^+^ T cells.^26^ Therefore, we assume that apoptosis, caspase-3 activation and pre-IL-16 processing are joint processes in human neutrophils.

Notably, in other studies, caspase-3 activation in the context of IL-16 processing was assessed by the appearance of the p20 fragment only. The p20 fragment of caspase-3 is a subunit of the p20:p12 heterodimer, which in a second step is autocatalytically cleaved into the p17 fragment, which forms the active p17:p12 caspase-3 heterodimer.^30^ However, recent publications also indicate a biological function of the intermediate heterodimer p20:p12, which is able to activate PKCδ, which then mediates the NF*κ*B-dependent synthesis of proinflammatory cytokines.^34^ Our data suggest that pre-IL-16 in neutrophils is cleaved by the apoptosis-associated p17:p12 heterodimer rather than the inflammation-associated p20:p12 heterodimer of caspase-3.

Most of the preformed proteins of neutrophils are packaged into granules during granulopoiesis. However, this process is questionable for IL-16 and MIF. Both cytokines lack signal peptides and therefore do not enter the ER and the Golgi apparatus. Consequently, they do not follow the conventional secretory pathway. Examining the intracellular localization of IL-16 and MIF, we found both cytokines to be localized in the cytosol of neutrophils, along with the calcium-binding S100A8 protein.^35^ This finding implies that IL-16 and MIF need to use unconventional pathways to be released, as described already for IL-1*β* ^36^ and FGF2.^37^ Testing a panel of cytokines, microbial constituents and glucocorticoids *in vitro*, we were not able find a stimulus that induced an active release of IL-16 and MIF by neutrophils (data not shown). Our data suggest that IL-16C and MIF are released passively by secondary necrotic neutrophils rather than actively by apoptotic neutrophils. As necrostatin-1, an inhibitor of necroptosis, did not affect the ratio of apoptotic and necrotic cells during a 72-h *in vitro* culture (data not shown), necroptosis is unlikely to have a role in spontaneous cell death of neutrophils. Accordingly, necrostatin-1 did not inhibit IL-16 release of neutrophils (data not shown). The predication that IL-16C and MIF are released by secondary necrotic neutrophils is strengthened by our results that after UV light irradiation and infection with *A. phagocytophilum,* IL-16 and MIF release is in line with enhanced secondary necrosis, but not with apoptosis. This result contradicts the finding of Daryadel *et al.*,^14^ who showed that MIF is released by apoptotic neutrophils dependent on ABC transporters, indicating an active release. However, other studies also describe the active secretion of IL-16 and MIF after S100A8,^38^ GM-CSF^38^ and C5a^39^ treatment. Repeating their protocols, we did not observe an elevated IL-16 and MIF release after treatment of our neutrophil preparations with S100A8, GM-CSF or C5a. On the contrary, as C5a and GM-CSF treatment decreased apoptosis and secondary necrosis after 24 h of incubation, we detected even less IL-16 and MIF in the supernatants of treated neutrophils (data not shown). Moreover, in studies by Riedemann *et al.* ^40^ and Daryadel *et al.* ^14^ the MIF concentrations in the supernatants of neutrophils are much higher than in our samples, reaching up to 8 ng/ml after 6 h of incubation of 5×10^6^ cells. Because the MIF concentrations measured by us using similar settings were always less than 50 pg/ml, we believe that the high concentrations in the other studies may be because of another isolation protocol for neutrophils, leading to more contamination with other immune cells.

As glucocorticoids are known to be potent inducers of MIF secretion from monocytes,^41^ we treated neutrophils with 1 *μ*M–0.1 fM dexamethasone or hydrocortisone for 12 h. As described previously, we observed an anti-apoptotic effect^42^ as well as a concomitant decrease in IL-16 and MIF release in the cells treated with higher concentrations of both glucocorticoids (data not shown).

In our hands, treatment with commonly used pro- and anti-inflammatory substances did not induce an active release of IL-16 and MIF by neutrophils; hence, the question arises of why these two cytokines exist as preformed cytokines when they are possibly only released during secondary necrosis. Although in the healthy organism apoptotic cells are immediately efferocytosed by professional phagocytes, this may not be the case during microbial and viral infections, as well as in autoimmune diseases. In this context, infection with HIV provides an interesting example. As H_2_O_2_ produced by neutrophils is viricidal to HIV-1,^43^ the virus impairs the function of H_2_O_2_ release and accelerates the apoptosis of neutrophils.^44^ Moreover, the HIV protein Nef inhibits the efferocytosis of apoptotic neutrophils by macrophages and monocytes.^45^ Ineffective clearance of apoptotic neutrophils will cause the transition to secondary necrosis. As IL-16C was shown to inhibit the replication of HIV-1,^46,47^ neutrophils may have found another way to reduce virus propagation by processing pre-IL-16 into IL-16C during apoptosis and releasing IL-16C during secondary necrosis. Moreover, impaired efferocytosis,^48,49^ as well as elevated expression levels of IL-16^50,51^ and MIF,^52,53^ have been observed to be connected with increased disease severity in autoimmune diseases such as rheumatoid arthritis and systemic lupus erythematosus. Although MIF has been shown to have a critical role in recruiting inflammatory immune cells in diseases such as rheumatoid arthritis,^54,55^ the biological role of IL-16 in autoimmune diseases still needs to be unraveled.

**Figure 6:**
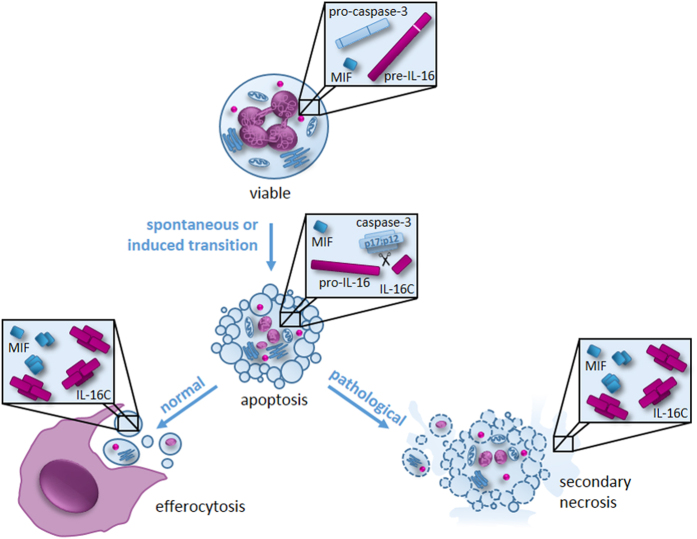
Schematic representation of the storage, processing and release of IL-16 and MIF in human neutrophils. MIF and IL-16 are preformed cytokines stored in the cytosol of human neutrophils. During apoptosis, the precursor of IL-16 (pre-IL-16) is processed into the biologically active IL-16C in a caspase-3-dependent manner. While under normal conditions, apoptotic cells are cleared by phagocytes, this process is impaired in infections and autoimmunity, leading to the release of MIF and IL-16C by secondary necrotic neutrophils.

In conclusion, the present study demonstrates that IL-16 and MIF are preformed cytokines present in the cytosol of mature human neutrophils that are released exclusively during secondary necrosis (Figure 6). Thus, it is plausible to assume that IL-16 and MIF contribute pivotally to the inflammatory immune reactions observed in diseases associated with defective clearance of senescent cells.

## Materials and Methods

### Isolation and culture of human neutrophils

Heparinized peripheral blood was obtained from healthy volunteers with written informed consent. All studies were approved by the ethics committee of the University of Lübeck (05–124). Granulocytes were isolated as described previously using discontinuous Percoll gradient centrifugation.^56^ Granulocyte purity and viability were >99.9%, as assessed by differential staining with Diff-Quik (Medion Diagnostics, Düdingen, Switzerland) and Annexin-V-FLUOS/PI staining. Only preparations with <3% eosinophils were used for the experiments.

All experiments were conducted using complete medium (RPMI 1640 medium (Sigma-Aldrich, Steinheim, Germany) supplemented with 10% FCS (Sigma-Aldrich), 50* μ*M 2-mercaptoethanol (Sigma-Aldrich), 4 mM L-glutamine (Biochrom, Berlin, Germany), 10 mM HEPES (Gibco Life Technologies, Paisley, Great Britain), 100 U/ml penicillin and 100 *μ*g/ml streptomycin (Biochrom)).

### Cytokine array

Freshly isolated neutrophils (1.5×10^7^) were resuspended in lysing buffer (1% Triton X-100 (Merck Millipore, Darmstadt, Germany), 10 *μ*g/ml aprotinin (Sigma-Aldrich), 10 *μ*g/ml leupeptin (Sigma-Aldrich) and 10 *μ*g/ml pepstatin A (Sigma-Aldrich) in PBS). After additional −70 °C freeze-and-thaw-disruption, the sample was centrifuged at 10 000×*g* for 5 min. The supernatants were collected and tested for preformed cytokines using the Proteome Profiler Array-Human Cytokine Array Panel A (R&D Systems) according to the manufacturer’s instructions.

### Immunofluorescence staining

Freshly isolated neutrophils were fixed (4% PFA), permeabilized (0.1% Triton X-100 in PBS) and blocked (10% BSA, 10% donkey serum). Human IL-16 C-terminal peptide antibody (Ab; R&D Systems, 1 : 25), human MIF Ab (R&D Systems, 1 : 25), anti-MRP Ab (Abcam, Cambridge, UK, 1 : 200) and anti-MMP9 Ab (Abcam, 1 : 50) were added and the cells were incubated at 4 °C for 12 h. The cells were washed and incubated with donkey anti-goat AlexaFluor 488 (Molecular Probes, Eugene, OR, USA, 1 : 200) for 1 h at room temperature. After washing and blocking (10% BSA, 10% goat serum), the cells were incubated with goat anti-rabbit AlexaFluor 555 (Cell Signaling Technologies, Danvers, MA, USA, 1 : 500) for 1 h at room temperature before being washed again. The cells were then mounted with ProLong Gold antifade reagent containing DAPI (Molecular Probes, Grand Island, NY, USA). Neutrophils stained only with the secondary Ab were used as a negative control. Images were obtained using an Olympus FV1000 confocal microscope (Olympus, Center Valley, PA, USA) with a ×60, NA=1.35 oil objective. Image capturing and analysis were performed using the Fluoview 2.1c software (Olympus). For colocalization studies, data sets were analyzed using the IMARIS software (Bitplane Scientific Software, South Windsor, CT, USA).

### Preparation of neutrophilic subcellular fractions

Granule subsets of neutrophils were purified applying a three-layer Percoll density gradient, as specified by Kjeldsen *et al.* ^57^ Aliquots of each fraction were assayed for the presence of marker proteins by ELISA.^58^ The fractions were pooled as follows: 1–7 were the *α*-band, 8–13 the *β*1-band, 14–17 the *β*2-band, 18–27 the *γ*-band and 28–39 the cytosol.

### Annexin-V-FLUOS/PI assay

For the Annexin-V-FLUOS/PI staining, cells were double-stained with Annexin-V-FLUOS (Roche, Mannheim, Germany) and PI (Sigma-Aldrich) according to the manufacturer’s instructions. The percentages of viable (Annexin-V-FLUOS^−^/PI^−^), apoptotic (Annexin-V-FLUOS^+^/PI^−^) and necrotic cells (Annexin-V-FLUOS^+^/PI^+^) were determined using a FACSCanto II and FACS Diva software (BD Biosciences, San Diego, CA, USA).

### Western blot analysis

For western blot analysis, neutrophils were lysed with 10% ice-cold trichloroacetic acid to prevent protein degradation by neutrophil proteases, as described previously.^59^

Proteins were separated by reducing SDS-PAGE, transferred to a nitrocellulose membrane and blocked for 30 min at room temperature with TBST containing 5% BSA. The membranes were then probed with primary Abs (human/mouse/rat MIF Ab, 1 : 800; human IL-16 C-terminal peptide Ab, 1 : 1333; human S100A8 Ab, 1 : 800 or human/mouse caspase-3 Ab, 1 : 400; purchased from R&D Systems) at 4 °C overnight. After washing, the membranes were probed with the corresponding secondary Abs (sheep IgG HRP-conjugated Ab, 1 : 1000; goat IgG HRP-conjugated Ab, 1 : 1000, R&D Systems) for 1 h at room temperature. After washing, the membranes were soaked with Immobilon Western chemiluminescent HRP substrate (Millipore, Billerica, MA, USA). Signals of binding Abs were detected with the Fusion FX7 chemiluminescence reader and quantified using the Bio1D software (Vilber Lourmat, Eberhardzell, Germany). Equal loading of the gel was determined by reprobing the membranes with GAPDH (14C10) rabbit HRP-conjugated Ab, 1 : 1000 (Cell Signaling Technologies).

### ELISA for IL-16 and MIF detection

Concentrations of human IL-16 and MIF in the supernatants were quantified using commercial ELISA kits (DuoSets, R&D Systems) according to the manufacturer’s instructions. According to the manufacturer’s information, the antibodies used in the human IL-16 DuoSet are directed against rhIL-16C (#AAC12732). Nevertheless, cross-reactivity with pre-IL-16 cannot be excluded, as the manufacturer did not test the detection of pre-IL-16. Assuming no resynthesis of pre-IL-16 during apoptosis, our own testing, using lysates (as prepared in the cytokine array) of 5×10^6^ freshly isolated and MACS-enriched apoptotic neutrophils, indicates a 3.7 times stronger recognition of IL-16C than of pre-IL-16 (mean 710.9 pg/ml *versus* mean 192.6 pg/ml, *n*=3, *P*=0.040 as calculated by *t*-test for paired samples).

### UV-light-induced apoptosis

To facilitate neutrophil apoptosis, 350 *μ*l of 7×10^6^ freshly isolated neutrophils were irradiated with 256 nm wavelength UV light (200–1600 mJ/cm^2^) using a Stratalinker (Stratagene, Heidelberg, Germany). Subsequently, the irradiated cells were incubated at a concentration of 5×10^6^ cells/ml in complete medium for 6 h at 37 °C.

### Preparation of cell-free *A. phagocytophilum* and co-incubation with neutrophils

The infection rate of *A. phagocytophilum*-exposed HL-60 cells was determined by counting the cells containing morulae in Diff-Quik-stained cytospin preparations. When ≥40% of the cells were found to be infected, they were used for the preparation of cell-free *A. phagocytophilum*, as described previously.^60^ Briefly, infected HL-60 cells were centrifuged at a speed of 250×*g* for 10 min, resuspended in 2 ml of PBS and passed through a 25-gauge needle 10–14 times. Cellular debris was removed by pelleting at 750×*g* for 10 min. The supernatant was collected and centrifuged at 2500×*g* for 15 min. *A. phagocytophilum* obtained in this way was immediately resuspended in neutrophil suspension and incubated for 6 h at 37 °C. For the infection of neutrophils, *A. phagocytophilum* derived from one infected HL-60 cell was used to infect one PMN.^31^

## Abbreviations

Ab: antibody
A. phago.: *Anaplasma phagocytophilum*
AU: arbitrary units
DAMPs: danger-associated molecular pattern molecules
IL-16: interleukin-16
LDH: lactate dehydrogenase
MIF: macrophage migration inhibitory factor
PI: propidium iodide.

## References

[bib1] Saverymuttu SH , Peters AM , Keshavarzian A , Reavy HJ , Lavender JP . The kinetics of 111indium distribution following injection of 111indium labelled autologous granulocytes in man. Br J Haematol 1985; 61: 675–685. 4084457 10.1111/j.1365-2141.1985.tb02882.x

[bib2] McCracken JM , Allen L-AH . Regulation of human neutrophil apoptosis and lifespan in health and disease. J Cell Death 2014; 7: 15–23. 25278783 10.4137/JCD.S11038 PMC4167320

[bib3] Silva MT . Secondary necrosis: the natural outcome of the complete apoptotic program. FEBS Lett 2010; 584: 4491–4499. 20974143 10.1016/j.febslet.2010.10.046

[bib4] Muñoz LE , Lauber K , Schiller M , Manfredi AA , Herrmann M . The role of defective clearance of apoptotic cells in systemic autoimmunity. Nat Rev Rheumatol 2010; 6: 280–289. 20431553 10.1038/nrrheum.2010.46

[bib5] Denecker G , Vercammen D , Steemans M , Vanden Berghe T , Brouckaert G , Van Loo G et al. Death receptor-induced apoptotic and necrotic cell death: differential role of caspases and mitochondria. Cell Death Differ 2001; 8: 829–840. 11526436 10.1038/sj.cdd.4400883

[bib6] Wu X , Molinaro C , Johnson N , Casiano CA . Secondary necrosis is a source of proteolytically modified forms of specific intracellular autoantigens: implications for systemic autoimmunity. Arthritis Rheum 2001; 44: 2642–2652. 11710720 10.1002/1529-0131(200111)44:11<2642::aid-art444>3.0.co;2-8

[bib7] Kolaczkowska E , Kubes P . Neutrophil recruitment and function in health and inflammation. Nat Rev Immunol 2013; 13: 159–175. 23435331 10.1038/nri3399

[bib8] Amulic B , Cazalet C , Hayes GL , Metzler KD , Zychlinsky A . Neutrophil function: from mechanisms to disease. Annu Rev Immunol 2012; 30: 459–489. 22224774 10.1146/annurev-immunol-020711-074942

[bib9] Sheshachalam A , Srivastava N , Mitchell T , Lacy P , Eitzen G . Granule protein processing and regulated secretion in neutrophils. Front Immunol 2014; 5: 448. 25285096 10.3389/fimmu.2014.00448 PMC4168738

[bib10] Borregaard N . Neutrophils, from marrow to microbes. Immunity 2010; 33: 657–670. 21094463 10.1016/j.immuni.2010.11.011

[bib11] Pellmé S , Mörgelin M , Tapper H , Mellqvist U-H , Dahlgren C , Karlsson A . Localization of human neutrophil interleukin-8 (CXCL-8) to organelle(s) distinct from the classical granules and secretory vesicles. J Leukoc Biol 2006; 79: 564–573. 16387844 10.1189/jlb.0505248

[bib12] Matzer SP , Baumann T , Lukacs NW , Röllinghoff M , Beuscher HU . Constitutive expression of macrophage-inflammatory protein 2 (MIP-2) mRNA in bone marrow gives rise to peripheral neutrophils with preformed MIP-2 protein. J Immunol 2001; 167: 4635–4643. 11591793 10.4049/jimmunol.167.8.4635

[bib13] Faurschou M , Borregaard N . Neutrophil granules and secretory vesicles in inflammation. Microbes Infect 2003; 5: 1317–1327. 14613775 10.1016/j.micinf.2003.09.008

[bib14] Daryadel A , Grifone RF , Simon H-U , Yousefi S . Apoptotic neutrophils release macrophage migration inhibitory factor upon stimulation with tumor necrosis factor-alpha. J Biol Chem 2006; 281: 27653–27661. 16861224 10.1074/jbc.M604051200

[bib15] Terebuh PD , Otterness IG , Strieter RM , Lincoln PM , Danforth JM , Kunkel SL et al. Biologic and immunohistochemical analysis of interleukin-6 expression* in vivo*. Constitutive and induced expression in murine polymorphonuclear and mononuclear phagocytes. Am J Pathol 1992; 140: 649–657. 1372159 PMC1886159

[bib16] Bliss SK , Butcher BA , Denkers EY . Rapid recruitment of neutrophils containing prestored IL-12 during microbial infection. J Immunol 2000; 165: 4515–4521. 11035091 10.4049/jimmunol.165.8.4515

[bib17] Brandt E , Woerly G , Younes AB , Loiseau S , Capron M . IL-4 production by human polymorphonuclear neutrophils. J Leukoc Biol 2000; 68: 125–130. 10914499

[bib18] Beil WJ , Weller PF , Peppercorn MA , Galli SJ , Dvorak AM . Ultrastructural immunogold localization of subcellular sites of TNF-alpha in colonic Crohn’s disease. J Leukoc Biol 1995; 58: 284–298. 7665984 10.1002/jlb.58.3.284

[bib19] Calafat J , Janssen H , Ståhle-Bäckdahl M , Zuurbier AE , Knol EF , Egesten A . Human monocytes and neutrophils store transforming growth factor-alpha in a subpopulation of cytoplasmic granules. Blood 1997; 90: 1255–1266. 9242560

[bib20] Schröder AK , von der Ohe M , Kolling U , Altstaedt J , Uciechowski P , Fleischer D et al. Polymorphonuclear leucocytes selectively produce anti-inflammatory interleukin-1 receptor antagonist and chemokines, but fail to produce pro-inflammatory mediators. Immunology 2006; 119: 317–327. 17067311 10.1111/j.1365-2567.2006.02435.x PMC1819575

[bib21] Borregaard N , Sørensen OE , Theilgaard-Mönch K . Neutrophil granules: a library of innate immunity proteins. Trends Immunol 2007; 28: 340–345. 17627888 10.1016/j.it.2007.06.002

[bib22] Flieger O , Engling A , Bucala R , Lue H , Nickel W , Bernhagen J . Regulated secretion of macrophage migration inhibitory factor is mediated by a non-classical pathway involving an ABC transporter. FEBS Lett 2003; 551: 78–86. 12965208 10.1016/s0014-5793(03)00900-1

[bib23] Zhang Y , Center DM , Wu DM , Cruikshank WW , Yuan J , Andrews DW et al. Processing and activation of pro-interleukin-16 by caspase-3. J Biol Chem 1998; 273: 1144–1149. 9422780 10.1074/jbc.273.2.1144

[bib24] Baier M , Bannert N , Werner A , Lang K , Kurth R . Molecular cloning, sequence, expression, and processing of the interleukin 16 precursor. Proc Natl Acad Sci USA 1997; 94: 5273–5277. 9144227 10.1073/pnas.94.10.5273 PMC24668

[bib25] Richmond J , Tuzova M , Cruikshank W , Center D . Regulation of Cellular Processes by Interleukin-16 in Homeostasis and Cancer. J Cell Physiol 2014; 229: 139–147. 23893766 10.1002/jcp.24441

[bib26] Wu DM , Zhang Y , Parada NA , Kornfeld H , Nicoll J , Center DM et al. Processing and release of IL-16 from CD4+ but not CD8+ T cells is activation dependent. J Immunol 1999; 162: 1287–1293. 9973381

[bib27] Liu Y , Cruikshank WW , O’Loughlin T , O’Reilly P , Center DM , Kornfeld H . Identification of a CD4 domain required for interleukin-16 binding and lymphocyte activation. J Biol Chem 1999; 274: 23387–23395. 10438516 10.1074/jbc.274.33.23387

[bib28] Lynch EA , Heijens CAW , Horst NF , Center DM , Cruikshank WW . Cutting edge: IL-16/CD4 preferentially induces Th1 cell migration: requirement of CCR5. J Immunol 2003; 171: 4965–4968. 14607889 10.4049/jimmunol.171.10.4965

[bib29] Scheel-Toellner D , Wang K , Craddock R , Webb PR , McGettrick HM , Assi LK et al. Reactive oxygen species limit neutrophil life span by activating death receptor signaling. Blood 2004; 104: 2557–2564. 15238425 10.1182/blood-2004-01-0191

[bib30] Han Z , Hendrickson EA , Bremner TA , Wyche JH . A sequential two-step mechanism for the production of the mature p17:p12 form of caspase-3 *in vitro*. J Biol Chem 1997; 272: 13432–13436. 9148968 10.1074/jbc.272.20.13432

[bib31] Sarkar A , Hellberg L , Bhattacharyya A , Behnen M , Wang K , Lord JM et al. Infection with Anaplasma phagocytophilum activates the phosphatidylinositol 3-Kinase/Akt and NF-κB survival pathways in neutrophil granulocytes. Infect Immun 2012; 80: 1615–1623. 22252875 10.1128/IAI.05219-11 PMC3318406

[bib32] Tecchio C , Micheletti A , Cassatella MA . Neutrophil-derived cytokines: facts beyond expression. Front Immunol 2014; 5: 1–7. 25374568 10.3389/fimmu.2014.00508 PMC4204637

[bib33] Gaudry M , Brégerie O , Andrieu V , El Benna J , Pocidalo MA , Hakim J . Intracellular pool of vascular endothelial growth factor in human neutrophils. Blood 1997; 90: 4153–4161. 9354686

[bib34] Kavanagh E , Rodhe J , Burguillos MA , Venero JL , Joseph B . Regulation of caspase-3 processing by cIAP2 controls the switch between pro-inflammatory activation and cell death in microglia. Cell Death Dis 2014; 5: e1565. 25501826 10.1038/cddis.2014.514 PMC4454160

[bib35] Hessianl PA , Edgeworth J , Hogg N . MRP-8 and MRP-14, two abundant Ca(2+)-binding proteins of neutrophils and monocytes. J Leukoc Biol 1993; 53: 197–204. 8445331

[bib36] Lopez-Castejon G , Brough D . Understanding the mechanism of IL-1*β* secretion. Cytokine Growth Factor Rev 2011; 22: 189–195. 22019906 10.1016/j.cytogfr.2011.10.001 PMC3714593

[bib37] Steringer JP , Müller H-M , Nickel W . Die molekulare Entschlüsselung unkonventioneller Sekretionsmechanismen. BIOspektrum 2014; 20: 400–403.

[bib38] Katano M , Okamoto K , Suematsu N , Kurokawa MS , Nakamura H , Masuko K et al. Increased expression of S100 calcium binding protein A8 in GM-CSF-stimulated neutrophils leads to the increased expressions of IL-8 and IL-16. Clin Exp Rheumatol 2011; 29: 768–775. 21961943

[bib39] Riedemann NC , Guo R-F , Gao H , Sun L , Hoesel M , Hollmann TJ et al. Regulatory role of C5a on macrophage migration inhibitory factor release from neutrophils. J Immunol 2004; 173: 1355–1359. 15240730 10.4049/jimmunol.173.2.1355

[bib40] Riedemann NC , Guo R-F , Gao H , Sun L , Hoesel M , Hollmann TJ et al. Regulatory role of C5a on macrophage migration inhibitory factor release from neutrophils. J Immunol 2004; 173: 1355–1359. 15240730 10.4049/jimmunol.173.2.1355

[bib41] Calandra T , Bernhagen J , Metz CN , Spiegel LA , Bacher M , Donnelly T et al. MIF as a glucocorticoid-induced modulator of cytokine production. Nature 1995; 377: 68–71. 7659164 10.1038/377068a0

[bib42] Ruiz LM , Bedoya G , Salazar J , García de OD , Patiño PJ . Dexamethasone inhibits apoptosis of human neutrophils induced by reactive oxygen species. Inflammation 2002; 26: 215–222. 12238564 10.1023/a:1019714618068

[bib43] Klebanoff SJ , Coombs RW . Viricidal effect of polymorphonuclear leukocytes on human immunodeficiency virus-1. Role of the myeloperoxidase system. J Clin Invest 1992; 89: 2014–2017. 1318327 10.1172/JCI115810 PMC295907

[bib44] Pitrak DL , Tsai HC , Mullane KM , Sutton SH , Stevens P . Accelerated neutrophil apoptosis in the acquired immunodeficiency syndrome. J Clin Invest 1996; 98: 2714–2719. 8981916 10.1172/JCI119096 PMC507735

[bib45] Torre D , Gennero L , Baccino FM , Speranza F , Biondi G , Pugliese A . Impaired macrophage phagocytosis of apoptotic neutrophils in patients with human immunodeficiency virus type 1 infection. Clin Diagn Lab Immunol 2002; 9: 983–986. 12204947 10.1128/CDLI.9.5.983-986.2002 PMC120074

[bib46] Idziorek T , Khalife J , Billaut-Mulot O , Hermann E , Aumercier M , Mouton Y et al. Recombinant human IL-16 inhibits HIV-1 replication and protects against activation-induced cell death (AICD). Clin Exp Immunol 1998; 112: 84–91. 9566794 10.1046/j.1365-2249.1998.00550.x PMC1904943

[bib47] Amiel C , Darcissac E , Truong MJ , Dewulf J , Loyens M , Mouton Y et al. Interleukin-16 (IL-16) inhibits human immunodeficiency virus replication in cells from infected subjects, and serum IL-16 levels drop with disease progression. J Infect Dis 1999; 179: 83–91. 9841826 10.1086/314550

[bib48] Shao W , Cohen PL . Disturbances of apoptotic cell clearance in systemic lupus erythematosus. Arthritis Res Ther 2011; 13: 1–7. 10.1186/ar3206 PMC3157636 21371352

[bib49] Ren Y , Tang J , Mok MY , Chan AWK , Wu A , Lau CS . Increased apoptotic neutrophils and macrophages and impaired macrophage phagocytic clearance of apoptotic neutrophils in systemic lupus erythematosus. Arthritis Rheum 2003; 48: 2888–2897. 14558095 10.1002/art.11237

[bib50] Lard LR , Roep BO , Verburgh CA , Zwinderman AH , Huizinga TWJ . Elevated IL-16 levels in patients with systemic lupus erythematosus are associated with disease severity but not with genetic susceptibility to lupus. Lupus 2002; 11: 181–185. 11999883 10.1191/0961203302lu176sr

[bib51] Lee S , Kaneko H , Sekigawa I , Tokano Y , Takasaki Y , Hashimoto H . Circulating interleukin-16 in systemic lupus erythematosus. Br J Rheumatol 1998; 37: 1334–1337. 9973160 10.1093/rheumatology/37.12.1334

[bib52] Kasama T , Ohtsuka K , Sato M , Takahashi R , Wakabayashi K , Kobayashi K . Macrophage migration inhibitory factor: a multifunctional cytokine in rheumatic diseases. Arthritis 2010; 2010: 106202. 22046508 10.1155/2010/106202 PMC3195319

[bib53] Ayoub S , Hickey MJ , Morand EF . Mechanisms of disease: macrophage migration inhibitory factor in SLE, RA and atherosclerosis. Nat Clin Pract Rheumatol 2008; 4: 98–105. 18235539 10.1038/ncprheum0701

[bib54] Santos LL , Fan H , Hall P , Ngo D , Mackay CR , Fingerle-Rowson G et al. Macrophage migration inhibitory factor regulates neutrophil chemotactic responses in inflammatory arthritis in mice. Arthritis Rheum 2011; 63: 960–970. 21452319 10.1002/art.30203 PMC3069137

[bib55] Gregory JL , Morand EF , McKeown SJ , Ralph JA , Hall P , Yang YH et al. Macrophage migration inhibitory factor induces macrophage recruitment via CC chemokine ligand 2. J Immunol 2006; 177: 8072–8079. 17114481 10.4049/jimmunol.177.11.8072

[bib56] Aga E , Katschinski DM , van Zandbergen G , Laufs H , Hansen B , Müller K et al. Inhibition of the spontaneous apoptosis of neutrophil granulocytes by the intracellular parasite Leishmania major. J Immunol 2002; 169: 898–905. 12097394 10.4049/jimmunol.169.2.898

[bib57] Kjeldsen L , Sengelov H , Borregaard N . Subcellular fractionation of human neutrophils on Percoll density gradients. J Immunol Methods 1999; 232: 131–143. 10618515 10.1016/s0022-1759(99)00171-4

[bib58] Udby L , Borregaard N . Subcellular fractionation of human neutrophils and analysis of subcellular markers. Methods Mol Biol 2007; 412: 35–56. 18453104 10.1007/978-1-59745-467-4_4

[bib59] Hellberg L , Samavedam UKSRL , Holdorf K , Hänsel M , Recke A , Beckmann T et al. Methylprednisolone blocks autoantibody-induced tissue damage in experimental models of bullous pemphigoid and epidermolysis bullosa acquisita through inhibition of neutrophil activation. J Invest Dermatol 2013; 133: 2390–2399. 23448878 10.1038/jid.2013.91

[bib60] Choi K-S , Park JT , Dumler JS . Anaplasma phagocytophilum delay of neutrophil apoptosis through the p38 mitogen-activated protein kinase signal pathway. Infect Immun 2005; 73: 8209–8218. 16299317 10.1128/IAI.73.12.8209-8218.2005 PMC1307085

