# Supplementary material for: Secondary necrotic neutrophils release interleukin-16C and macrophage migration inhibitory factor from stores in the cytosol
Source: Cell Death Discov. 2015 Nov 30;1:15056–. doi: 10.1038/cddiscovery.2015.56 (PMC4979515; doi:10.1038/cddiscovery.2015.56)
Supplement: Supplementary Information [file cddiscovery201556-s1.doc]

**Supplementary information**

As other studies described only a single band at 80 kDa for pre-IL-16, we were concerned about the finding of a double band at 75-80 kDa. Moreover, we did not observe this double band in the western blot analysis of the subcellular fractions (Figure 2B). Because we used trichloroacetic acid (TCA) for the preparation of whole lysates but not for the samples of the subcellular fractions, we compared the influence of TCA on the pre-IL-16 bands detected by western blot analysis. This analysis revealed that the depicted double band is a consequence of TCA lysis, because samples prepared without TCA showed only a single band at 80 kDa. We use TCA to lyse neutrophils and precipitate the containing proteins in an acidic environment, which inactivates the strong endogenous proteases contained in neutrophils. As conventional lysing protocols do not lead to the complete inactivation of neutrophil proteases, many cellular proteins are exposed to proteolytic degradation. Indeed, in the samples prepared without TCA, the reference protein GAPDH is fully degraded (Supplementary information and Supplementary Figure 1). Notably, pre-IL-16 in the same sample appeared not to be susceptible to degradation.
