# Supplementary figures and images for: Secondary necrotic neutrophils release interleukin-16C and macrophage migration inhibitory factor from stores in the cytosol
Source: Cell Death Discov. 2015 Nov 30;1:15056–. doi: 10.1038/cddiscovery.2015.56 (PMC4979515; doi:10.1038/cddiscovery.2015.56)

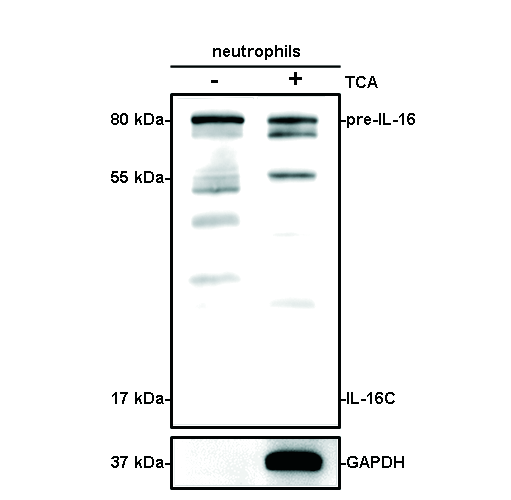

Supplement: Supplementary Figure 1 [file cddiscovery201556-s2.tiff]
